# Early and late effects of pharmacological ALK inhibition on the neuroblastoma transcriptome

**DOI:** 10.18632/oncotarget.22423

**Published:** 2017-11-06

**Authors:** Shana Claeys, Geertrui Denecker, Robrecht Cannoodt, Candy Kumps, Kaat Durinck, Frank Speleman, Katleen De Preter

**Affiliations:** ^1^ Center for Medical Genetics, Ghent University, Ghent, Belgium; ^2^ Cancer Research Institute Ghent, Ghent University, Ghent, Belgium; ^3^ Bioinformatics Institute Ghent From Nucleotides to Networks, Ghent, Belgium; ^4^ Data Mining and Modelling for Biomedicine group, VIB Inflammation Research Center, Ghent, Belgium; ^5^ Department of Respiratory Medicine, Ghent University, Ghent, Belgium; ^6^ Department of Uro-Gynaecology, Ghent University Hospital, Ghent, Belgium

**Keywords:** neuroblastoma, ALK, ALK inhibition, ALK signaling, dynamic

## Abstract

**Background:**

Neuroblastoma is an aggressive childhood malignancy of the sympathetic nervous system. Despite multi-modal therapy, survival of high-risk patients remains disappointingly low, underscoring the need for novel treatment strategies. The discovery of *ALK* activating mutations opened the way to precision treatment in a subset of these patients. Previously, we investigated the transcriptional effects of pharmacological ALK inhibition on neuroblastoma cell lines, six hours after TAE684 administration, resulting in the 77-gene ALK signature, which was shown to gradually decrease from 120 minutes after TAE684 treatment, to gain deeper insight into the molecular effects of oncogenic ALK signaling.

**Aim:**

Here, we further dissected the transcriptional dynamic profiles of neuroblastoma cells upon TAE684 treatment in a detailed timeframe of ten minutes up to six hours after inhibition, in order to identify additional early targets for combination treatment.

**Results:**

We observed an unexpected initial upregulation of positively regulated MYCN target genes following subsequent downregulation of overall MYCN activity. In addition, we identified adrenomedullin (ADM), previously shown to be implicated in sunitinib resistance, as the earliest response gene upon ALK inhibition.

**Conclusions:**

We describe the early and late effects of ALK inhibitor TAE684 treatment on the neuroblastoma transcriptome. The observed unexpected upregulation of ADM warrants further investigation in relation to putative ALK resistance in neuroblastoma patients currently undergoing ALK inhibitor treatment.

## INTRODUCTION

Neuroblastoma (NB) is a childhood cancer arising from malignant transformation of cells from the sympatho-adrenergic lineage of the developing neural crest [1] and is characterized by a large clinical heterogeneity, including patients with tumours that spontaneously regress as well as patients with metastasis and refractory disease despite intensive therapy regimens [2]. For these high-risk neuroblastoma cases, a better understanding of the genes and pathways that are involved in disease development and progression is currently fuelling the identification of new molecular targets for therapy [3].

Overall, given the low frequency of mutations in neuroblastoma, options for targeted therapy are relatively limited. However, the recurrent somatic mutations of the tyrosine kinase receptor *ALK* identified in 8-10% of primary neuroblastoma tumours [4–8] and the emergence of *ALK* mutations at relapse mark ALK as an important novel drug target [9, 10]. Several orally available small molecule ALK inhibitors have been developed and successfully applied in patients with other ALK mutant tumour entities, so-called ALKoma’s, most notably a subset of lung cancers [11–17]. Several of these small molecule ALK inhibitors have recently gone into phase 1 clinical trials for patients with refractory neuroblastoma, inflammatory myofibroblastic tumour, non-small-cell lung cancer (NSCLC) and anaplastic large-cell lymphoma (ALCL) [18, 19].

Despite the promising therapeutic potential of ALK inhibitors, an important drawback is the rapid occurrence of resistance due to escape mechanisms including secondary mutations or activation of other kinase signaling pathways [17, 20–30]. A better understanding of the downstream ALK signaling cascades can inspire the development of more rationally designed combinatorial treatment approaches. In a previous study, ALK downstream signaling was characterized by in depth transcriptome analysis of neuroblastoma cells treated for six hours with the ALK inhibitor TAE684, resulting in the 77-gene ALK signature and the identification of a negative MAPK feedback loop and of RET as ALK activated target gene [31]. Moreover, we generated transcriptome data between 10 minutes and 6 hours after pharmacological ALK inhibition to show that our established 77-gene signature is gradually decreasing starting from 2 hours after the treatment. Here, we further explore this dynamic transcriptome data generated upon treatment with the ALK inhibitor TAE684 in order to look in more detail into the dynamics of early transcriptional responses following ALK inhibition to improve our understanding on downstream ALK signaling and identify novel targets for combination therapy. To this end, we performed differential gene expression analysis at different time points following TAE684 treatment of the neuroblastoma cell line CLB-GA with an activating ALK^R1275Q^ mutation.

## RESULTS AND DISCUSSION

### MAPK, PI_3_K, RET and MYC(N) signaling pathways are downregulated starting from 1 to 2 hours after TAE684 treatment

In order to gain more insights into the early dynamics of transcriptional changes after pharmacological inhibition of ALK signaling in neuroblastoma cells, the neuroblastoma cell line CLB-GA, which harbours an ALK^R1275Q^ mutation, was treated with the ALK inhibitor TAE684 and DMSO as control. While TAE684 did not go into clinical trials, TAE684 is a valid ALK-inhibitor as we previously showed that the transcriptional responses for this drug and the more clinically relevant ones, such as crizotinib, LDK378 and X396, are very similar [31]. RNA expression profiling was performed on cells harvested 10 and 30 minutes, 1, 2, 4 and 6 hours after treatment (Supplementary Figure 1).

**Figure 1:**
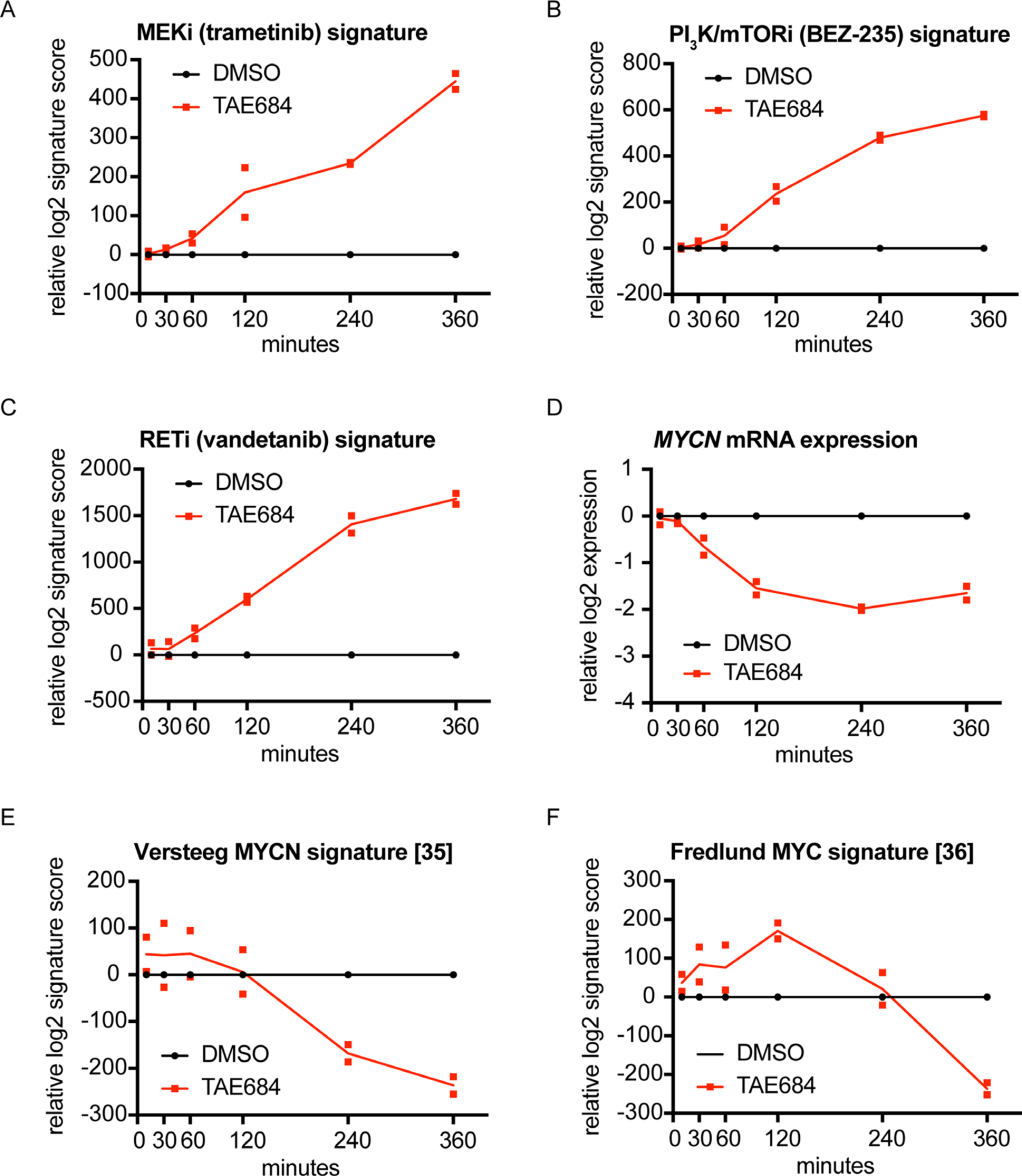
Signature score analysis for MAPK, PI_3_K, RET and MYC(N) signaling pathways, downregulated starting from 1 or 2 hours after TAE684 treatment (**A**–**E**) MEK inhibitor (trametinib) (A), PI_3_K/mTOR inhibitor (BEZ-235) (B) and RET inhibitor (vandetanib) (C) signatures are upregulated from 1 or 2 hours after treatment of the CLB-GA cell line with 320 nM TAE684, while *MYCN* mRNA expression levels (D) are downregulated from 1 hour after treatment and the MYCN activity score from Valentijn *et al.* [35] (E) and the MYC signature score from Fredlund *et al.* [36] (**F**) from respectively 2 and 4 hours after pharmacological ALK blockade. Log2 transformed ratios of expression levels of TAE684 treated vs DMSO control samples are plotted.

Our previously established 77-gene ALK signature [31] significantly decreases from 2 hours after ALK inhibitor treatment, as shown earlier by Lambertz *et al.* [31]. In this study, we evaluated the dynamic regulation of known ALK downstream pathways, i.e. MAPK, PI_3_K, RET and MYC/MYCN signaling pathways [4–7, 31–34], by calculating signature activity scores for drugs specifically blocking these pathways. These drug signature scores summarize the transcriptional response of the components of a given signaling pathway upon pharmacological pathway inhibition. Our data confirm ALK regulation of these pathways and furthermore show that MAPK, PI_3_K and RET pathway activity is decreased as early as 1 to 2 hours after the start of TAE684 treatment, as the corresponding inhibitor scores become activated at those time points (Figure 1A–1C; Supplementary Figures 2A–2C, 3A–3C). Furthermore, we observed that *MYCN* expression levels significantly drop 1 hour after the treatment (*p* = 0.0015) (Figure 1D; Supplementary Figure 2D). As expected, MYC(N) activity scores [35, 36] follow a delayed response at later time points (Figure 1E, 1F; Supplementary Figures 2E, 2F, 3D, 3E). These results are in line with the previously established regulatory role on *MYCN* transcription initiation for ALK, in addition to its effect on MYCN activity via phosphorylation [32, 34, 37–39].

**Figure 2:**
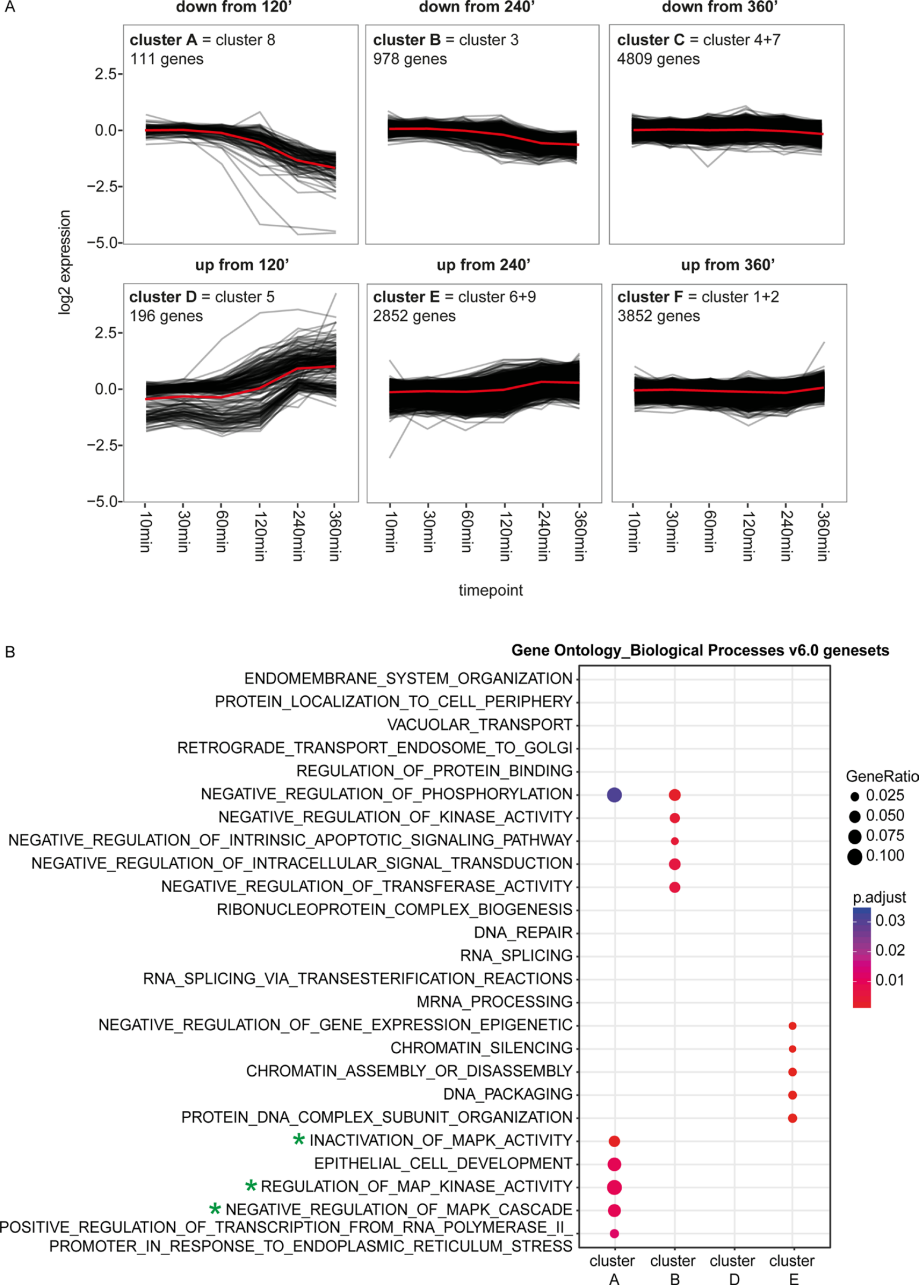
Functional characterization of the genes in the clusters with the MSigDB ‘c5 Gene Ontology (GO) Biological Process Ontology (BP) v6.0’ (**A**) The cluster plots represent the dynamic pattern of the expression of the genes belonging to 1 of the 6 clusters. The mean of the ratio of the expression in the TAE684 vs DMSO treated sample is plotted for each gene. The red lines show the average dynamic pattern of the expression of the genes belonging to these clusters, calculated by the average of the mean of the ratio of the expression in the TAE684 vs DMSO treated sample. (**B**) Plot showing the MSigDB ‘c5 Gene Ontology (GO) Biological Process Ontology (BP) v6.0’ genesets that are enriched in at least one of the four clusters, which are showing a clear dynamic pattern (clusters A, B, D, E). The size of each node corresponds to the number of genes overlapping between the cluster and the gene set and the colour represents the adjusted *p*-value of the enrichmen*t* test. Green stars indicate the genesets related to the MYC(N), KRAS-MAPK, PI_3_K/mTOR pathways.

To identify other dynamically regulated pathways upon ALK inhibition, functional enrichment analysis was performed on a set of k-means clusters of genes with similar transcriptional responses over the timeframe. After k-means clustering with 9 centers, as defined by the elbow method, we combined clusters with similar expression patterns over time yielding a total of 6 different clusters (Figure 2A; Supplementary Figures 4A, 4B, 5A). Moreover, 4 of these clusters, namely cluster A, B, D and E, are showing a clear dynamic pattern, with a change in expression starting at 120 or 240 minutes and increasing over time, while the other 2 are showing a more modest pattern over time with only a small change at the latest time point (Figure 2A, Supplementary Figure 5A). Functional characterization of these 4 clusters through evaluating the enrichment for gene ontology terms (MSigDB ‘c5 Gene Ontology (GO) Biological Process Ontology (BP) v6.0’), oncogenic signatures (MSigDB ‘c6 Oncogenic Signatures v5.0’) and pathways of the Reactome Database [40, 41] confirmed that these clusters consist of MYC, MAPK or mTOR targets and are involved in regulation of the MAPK cascade, in keeping with the downregulation of the MYC(N), PI_3_K-AKT-mTOR and MAPK pathway (Figure 2B; Supplementary Figure 5B, 5C). Moreover, cluster E is also enriched for genesets linked to epigenetic processes, regulating amongst others chromatin structure and gene expression (Figure 2B; Supplementary Figures 5B, 5C).

Taken together, this analysis provides insights into the dynamics of inactivation of ALK driven downstream pathways in ALK mutant neuroblastoma cells upon ALK inhibition and validates the applicability of the presented dataset to further scrutinize early temporal effects of ALK inhibition.

### MYC(N) signaling is upregulated immediately following ALK inhibition

**Figure 3:**
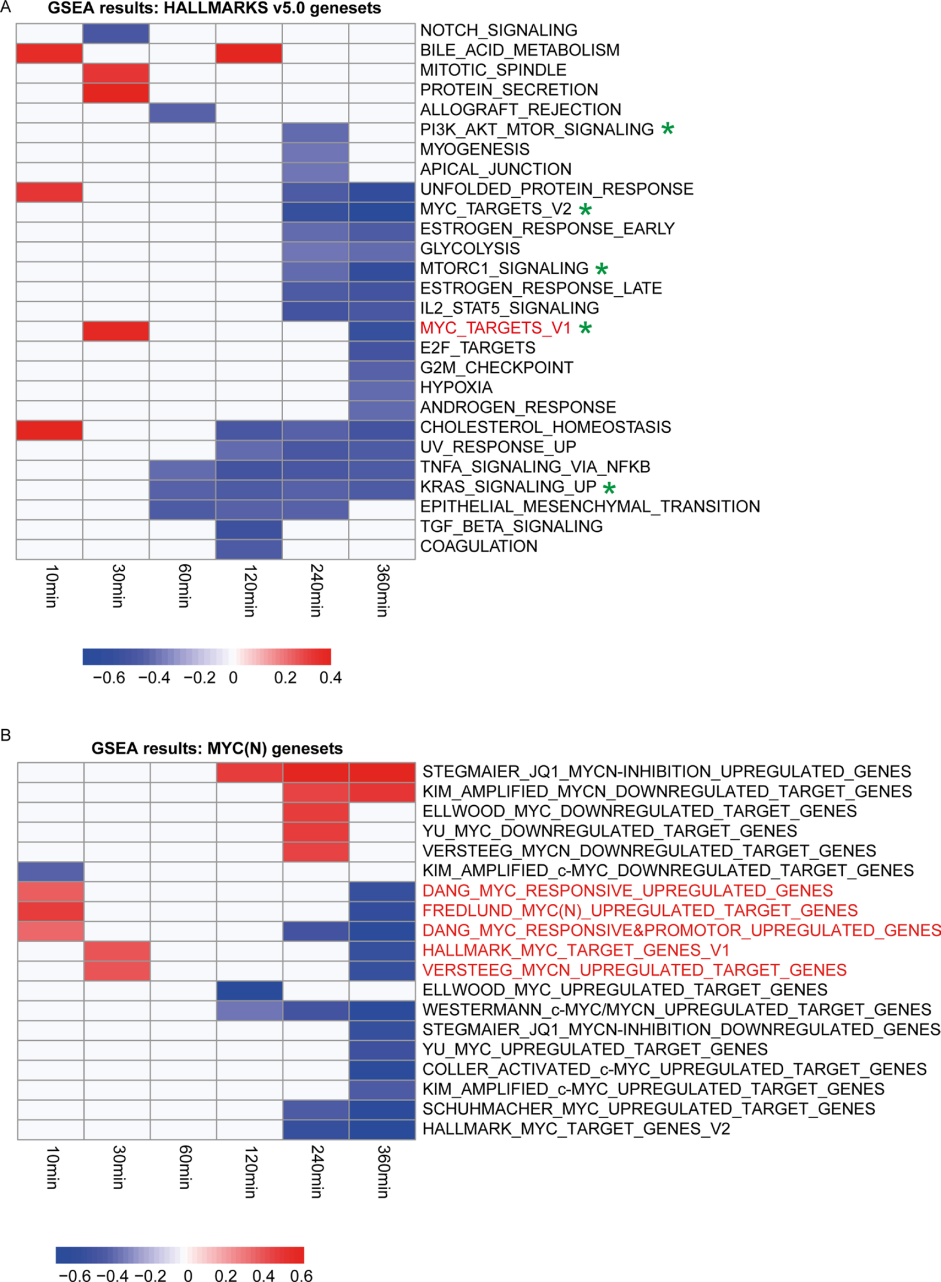
Gene Set Enrichment Analysis (GSEA) with the hallmarks and the MYC(N) signaling genesets shows that MYC(N) signaling is upregulated immediately following ALK inhibition (**A** and **B**) Heatmap showing the genesets of the ‘Hallmarks v5.0’ genesets from MSigDB (A) and an in house compiled gene set collection containing all MYC target genesets from this hallmark catalogue as well as publically available MYC(N) activity or target signatures [35, 60–66] (B), that are enriched among the genes upregulated after ALK inhibition (positively enriched) (red) or that are enriched among the genes downregulated after ALK inhibition (negatively enriched) (blue) according to GSEA (with FDR < 0.1) [41]. Indicated in red are the genesets that are upregulated at earlier and downregulated at the later time points following ALK inhibition. Green stars indicate the genesets related to the MYC(N), KRAS-MAPK, PI_3_K/mTOR pathways.

To functionally scrutinize the transcriptional changes upon ALK inhibitor treatment, Gene Set Enrichment Analysis (GSEA [42]) was performed using the ‘Hallmarks v5.0’ genesets from the Molecular Signatures Database (MSigDB) (software.broadinstitute.org/gsea/msigdb) at the different time points. This analysis further validates that the signaling pathways KRAS (MAPK) and PI_3_K-mTOR are significantly repressed (Figure 3A). Remarkably, one of the MYC genesets (HALLMARK_MYC_TARGETS_1) is significantly enriched for genes upregulated 30 minutes after treatment and significantly enriched for genes downregulated 6 hours after pharmacological ALK blockade (Figure 3A, indicated in red). Moreover, we observed a significant increase in the Fredlund MYC signature [36] 2 hours after ALK inhibition (*p* = 0.0038) (Figure 1F, Supplementary Figure 2F). Therefore, we also performed GSEA on other MYC(N) activity signatures and confirmed for 4 other genesets an initial upregulation (at 10 or 30 minutes after ALK inhibitor treatment) followed by a downregulation (from 2 hours after ALK inhibitor treatment) (Figure 3B). The observation of this initial upregulation of MYCN activity scores is intriguing and needs further investigation.

### Adrenomedullin (ADM) is the earliest upregulated gene upon pharmacological ALK inhibition of NB cells

In order to identify the set of early and late genes that significantly change transcriptionally, differential gene expression analysis comparing TAE684 treated cells with DMSO treated controls was performed at each time point. This analysis revealed that no significant transcriptional changes occur as early as 10 or 30 minutes after treatment (using adjusted *p*-value (False Discovery Rate (FDR)) < 0.05), while starting from 2 hours after treatment an increasing number of significantly, differentially expressed genes are identified (Supplementary Table 1). Our observations are in line with these from Lai *et al.* [43], who have shown that transcriptional events occur between 2 and 4 hours after inhibition of a receptor tyrosine kinase.

**Figure 4:**
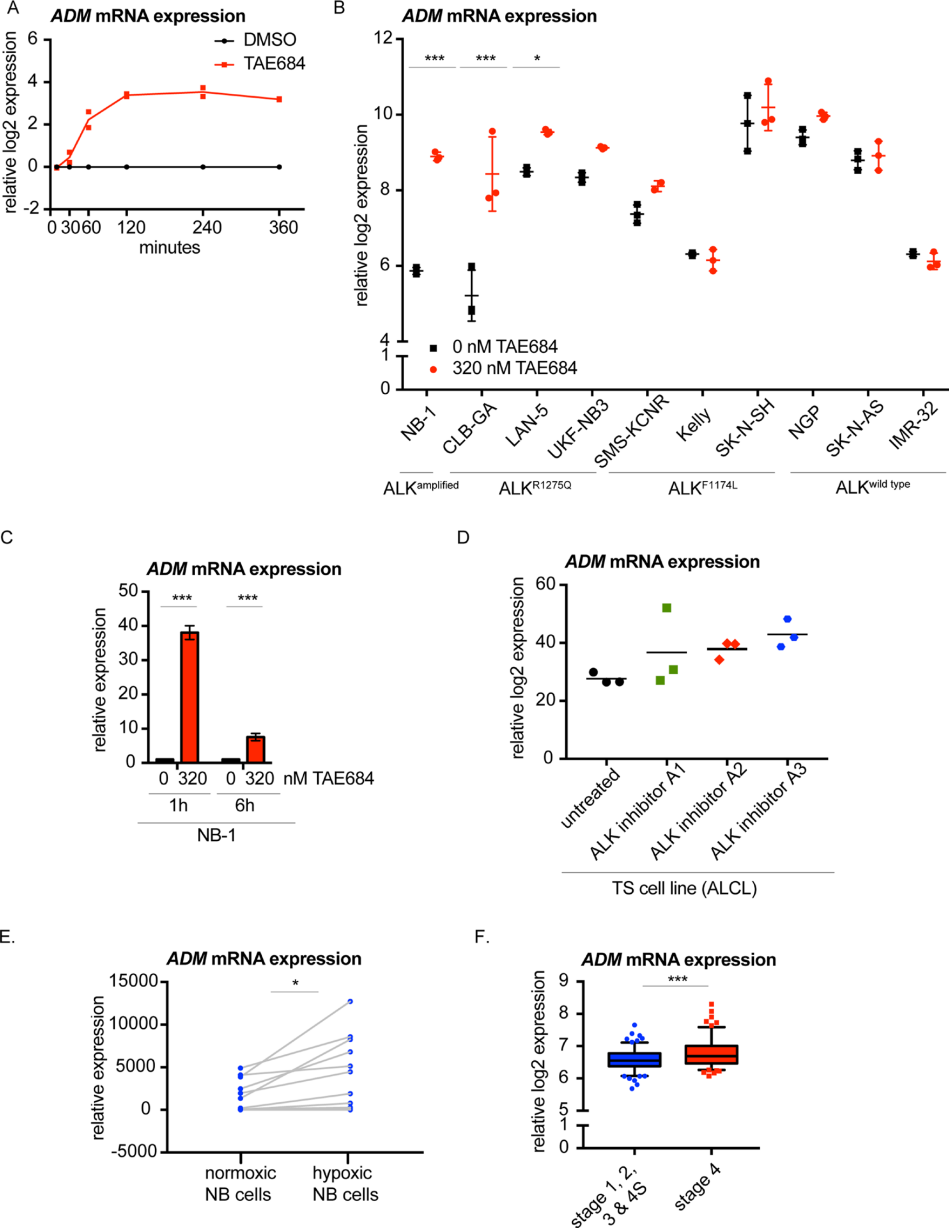
Adrenomedullin (ADM) is the earliest upregulated gene upon pharmacological ALK inhibition of NB cells (**A**) *ADM* mRNA is significantly upregulated starting from 1 hour after TAE684 treatment of the CLB-GA cell line. Ratios of log2 transformed expression levels of TAE684 treated vs DMSO control samples are plotted. (**B**) *ADM* mRNA expression levels shown in several ALK wild type (NGP, SK-N-AS, IMR-32) and ALK mutant (NB-1, CLB-GA, LAN-5, UKF-NB3, SMS-KCNR, Kelly, SK-N-SH) cell lines treated for 6 hours with 0.32 μM TAE684 or DMSO. (**C**)*ADM* mRNA expression levels are upregulated in NB cell line NB-1 (ALK^amp^) 1 hour and 6 hours after treatment with 0.32 μM TAE684 compared to the DMSO control. (**D**) *ADM* expression levels are upregulated 6 hours upon ALK inhibition with three different ALK inhibitors in the ALCL cell line TS. (**E**) *ADM* mRNA expression levels are upregulated in 11 NB cell lines under hypoxic conditions. (**F**) *ADM* expression levels are higher in stage 4 primary neuroblastoma tumours versus neuroblastoma tumours of stage 1, 2, 3 and 4S. Boxplots represent mean ± 95% confidence interval. Statistical analyses: unpaired one-way ANOVA with Bonferroni correction (B. & C. & D.), paired *t*-test (E.) and unpaired *t*-test (F.). ^*^P < 0.05, ^**^P < 0.01, ^***^P < 0.001.

Interestingly, one gene showed significant differential upregulation 1 hour post-TAE684 treatment, i.e. adrenomedullin (*ADM*) (Figure 4A; Supplementary Figure 6A). Interestingly, using second- and third-generation ALK inhibitors [31], the upregulation of *ADM* upon ALK inhibition was confirmed in CLB-GA and Kelly (Supplementary Figure 6B, 6C). Moreover, we could validate *ADM* upregulation 6 hours after treatment with TAE684 in more NB cell lines with an ALK amplification, an ALK^R1275Q^ or ALK^F1174L^ mutation (Figure 4B). Furthermore, the early upregulation of *ADM* was validated in the cell line NB-1 upon pharmacological ALK inhibition with TAE684 (Figure 4C), as the increase was already detectable 1 hour after the treatment. In addition, *ADM* expression levels are also significantly induced in the CLB-GA cell line, following PI_3_K/mTOR inhibitor BEZ-235, MEK antagonist trametinib and RET repressor vandetanib treatment [31] (Supplementary Figure 6D). Interestingly, *ADM* upregulation was also observed in the Anaplastic Large Cell Lymphoma (ALCL) cell line TS treated with three different ALK inhibitors for 6 hours [44] (Figure 4D).

### Adrenomedullin is upregulated under hypoxic conditions in neuroblastoma

As ADM is known to be induced under hypoxia [45–51], we investigated publically available transcriptome data from hypoxic versus normoxic neuroblastoma cells [52] and confirmed *ADM* upregulation under hypoxic conditions in these cells (Figure 4E). Of further interest, *ADM* is higher expressed in more aggressive neuroblastoma tumours (stage 4 versus other stages) (Figure 4F).

Furthermore, the observed *ADM* upregulation is intriguing and has a potentially important translational consequence. In addition to ADM activation upon hypoxia [45–51], ADM was also shown to act as growth factor, prevent apoptosis-mediated cell death, increase tumour cell motility and metastasis and induce angiogenesis in various cancer types [47–49, 51, 53–59]. Of further importance, it has been reported that hypoxia, which can activate ADM [45–51], induces resistance to ALK inhibitors in non-small cell lung cancer [60] and that ADM expression levels are increased in tyrosine kinase inhibitor sunitinib resistant renal cell carcinoma [61]. In view of these observations, we evaluated whether combining an adrenomedullin receptor antagonist (ADM22-52) [61] together with TAE684 would sensitize NB cells to ALK inhibition. No additional effects on decrease in cell viability were observed (data not shown). This observation could be due to the fact that elevated *ADM* levels have no effect on sensitivity for ALK inhibitors in neuroblastoma cells. Alternatively, given that the cell lines tested are very sensitive to the inhibitor with immediate strong and massive apoptotic effects, under these conditions elevated *ADM* levels may have no immediate effects on cell viability, while in a later stage in cells rendered resistant to ALK inhibition, combined exposure to both ALK inhibition and the ADM inhibitor might have effect.

## MATERIALS AND METHODS

### CLB-GA time series dataset

The CLB-GA time series dataset has been described before [31]. The neuroblastoma CLB-GA cell line (ALK^R1275Q^) was cultured in RPMI-1640 medium (Invitrogen), supplemented with fetal bovine serum (10%), kanamycin (100 μg/ml) penicillin/streptomycin (100 IU/ml), L-glutamine (2 mM) and HEPES (25 mM) (Life Technologies), and maintained at 37°C in a 5% C02-humidified environment. At different time points (10, 30 minutes, 1, 2, 4 and 6 hours) after treatment with 0.32 μM of the small molecule ALK inhibitor NVP-TAE684 (hereafter TAE684) (SelleckChem, S1108) or DMSO (VWR) as solvent control, cells were harvested and RNA quality was analysed using Experion (Bio-Rad) prior to gene-expression profiling. Samples were labelled and hybridized to the Sureprint G3 human GE 8x60K microarrays (Agilent Technologies), according to the manufacturer’s guidelines and starting from 200 ng RNA. The data was normalized with the vsn method in R version 3.3.3 (packages vsn and limma). The R package BioMart was used to annotate gene names to their corresponding probes. Probes detecting at least a two-fold higher expression than the negative control probes of the array in at least 60% of the DMSO or TAE samples, were selected as background correction. Data can be accessed through ArrayExpress (accession number E-MTAB-3206) [31].

### Signature score analysis

To establish MAPK, PI_3_K/mTOR and RET inhibitor signatures, we used published gene expression profiling data (ArrayExpress accession number E-MTAB-3206) [31]. This dataset contains expression profiling data of the CLB-GA cell line 6 hours after treatment with MAPK inhibitor trametinib, the dual PI_3_K/mTOR repressor BEZ-235 or RET antagonist vandetanib and DMSO as control. Using the limma R-package, differential expression analysis was performed comparing the DMSO-control and the inhibitor treated samples. The established signatures consist of the differentially expressed genes with adjusted *p*-value (False Discovery Rate (FDR)) < 0.05. In addition, a published MYCN and a published MYC signature were used to establish the MYC(N) pathway activity [35, 36]. Signatures score analysis was conducted using a rank-scoring algorithm as described previously [31, 36]. For each time point and each duplicate separately, both the ratio between the TAE684 treated sample and his corresponding control sample (Figure 1) as the absolute values (Supplementary Figure 2) were plotted using GraphPad Prism 7.

### Gene set enrichment analysis

Gene set enrichment analysis (GSEA [42]) was performed using the MSigDB ‘Hallmarks v5.0’ gene sets (software.broadinstitute.org/gsea/msigdb) and an in house compiled gene set catalogue containing all MYC target genesets from this hallmark catalogue as well as publically available MYC(N) activity or target signatures [35, 36, 62–67]. The genesets, showing positively or negatively enrichment at minimum one time point and with a FDR < 0.1 are plotted in a heatmap. Data was plotted as the mean of the ratio of the TAE684 treated sample and his corresponding control sample at every time point.

### Cluster analysis and pathway enrichment analysis in the clusters

Using the Bayesian Information Criterion (BIC) and the elbow method, 9 was defined as the optimal number of clusters in the dataset (Supplementary Figure 4A, 4B). K-means clustering was performed on the expression level ratios of TAE684/DMSO samples for each time point in R (with k = 9). However, visual inspection of the expression patterns of the genes in the clusters showed that some clusters have a similar pattern over time (clusters 1 and 2, 4 and 7, 6 and 9) (Supplementary Figure 4B) and were therefore merged, ending with 6 final clusters (Supplementary Figure 5A). For every cluster, the average response of the genes was plotted as a line (red line). The 4 clusters that are showing a clear dynamic pattern over time, were screened for MSigDB ‘c5 Gene Ontology (GO) Biological Process Ontology (BP) v6.0’ (software.broadinstitute.org/gsea/msigdb), MSigDB ‘c6 Oncogenic Signatures v5.0’ and overrepresented Reactome pathways [40, 41] using the R-packages clusterProfiler and Reactome Pathway Analysis [68, 69].

### Differential expression analysis

At every time point, differential expression analysis was performed using the R package limma, comparing the DMSO-control and the TAE684-treated sample. The duplicate experiments, independently generated at different time points, were set as blocking factor. Significantly differentially expressed genes refer to those with an adjusted *p*-value (FDR) < 0.001. Results are shown in Supplementary Table 2.

### Analysis of ADM expression levels after ALK inhibition using qPCR

*ADM* expression levels were measured in CLB-GA (ALK^R1275Q^) and Kelly (ALK^F1174L^) cells that were treated for 6 hours with DMSO as control solvent and 0.09 μM of ALK inhibitor, PF06463922 acetate (Sigma-Aldrich, PZ0271) (dissolved in sterile DMSO and diluted to the final concentration in culture medium) and in NB-1 (ALK^amp^) cells that were treated for 1 and 6 hours with DMSO as control solvent and 0.32 μM of TAE684 (dissolved in sterile DMSO and diluted to the final concentration in culture medium).

RNA extraction, cDNA synthesis and RT-qPCR of these samples was performed as described earlier [31]. The Cq-values for *ADM* expression were normalized with data of at least two reference genes (*TBP, YWHAZ, B2M, HPRT1* and *SDHA*) (primer sequences: Supplementary Table 2) using qBasePlus software (Biogazelle).

### Evaluation of ADM expression in public mRNA expression datasets

*ADM* expression levels were evaluated in (1) CLB-GA cells treated with 0.2 μM LDK378, 0.32 μM TAE684, 0.06 μM X396, 0.5 μM crizotinib, 0.05 μM trametinib (MEK-inhibitor), 0.5 μM BEZ-235 (dual PI_3_K/mTOR inhibitor) and 9.5 μM vandetanib for 6 hours (ArrayExpress accession number E-MTAB-3206) [31], (2) 10 NB cell lines treated with 0.32 μM TAE684 for 6 hours (ArrayExpress accession number E-MTAB-3205) [31], (3) the ALCL TS cell line treated with three different ALK inhibitors for 6 hours (GEO accession number GSE6184) [44], (4) 11 NB cell lines that were cultured under normoxic and hypoxic conditions (1% O_2_ instead of 20%) for 18 hours (GEO accession number GSE17714) [52] and (5) a cohort of 283 neuroblastoma tumour samples (GEO accession number GSE85047).

### Statistical analyses

Statistical significance was calculated with GraphPad Prism7 by unpaired one-way ANOVA with Bonferroni correction when comparing more than two unmatched groups, while (un)-paired *t*-test was chosen when comparing two groups.

## CONCLUSIONS

In summary, dynamic expression profiling following ALK inhibition of ALK mutated neuroblastoma cells revealed (1) unexpected early *ADM* upregulation with potential implications for design of more effective ALK targeted therapy, (2) an initial increase of MYC(N) activity immediately after ALK inhibition and (3) confirmed inhibition after 1 to 2 hours of the ALK downstream MAPK, PI_3_K-AKT, RET and MYC(N) pathways.

## SUPPLEMENTARY MATERIALS FIGURES AND TABLES




