# Supplementary material for: Early and late effects of pharmacological ALK inhibition on the neuroblastoma transcriptome
Source: Oncotarget. 2017 Nov 6;8(63):106820–32. doi: 10.18632/oncotarget.22423 (PMC5739776; doi:10.18632/oncotarget.22423)
Supplement: Supplementary file 1 [file oncotarget-08-106820-s001.pdf]

Early and late effects of pharmacological ALK inhibition on the neuroblastoma transcriptome

SUPPLEMENTARY MATERIALS

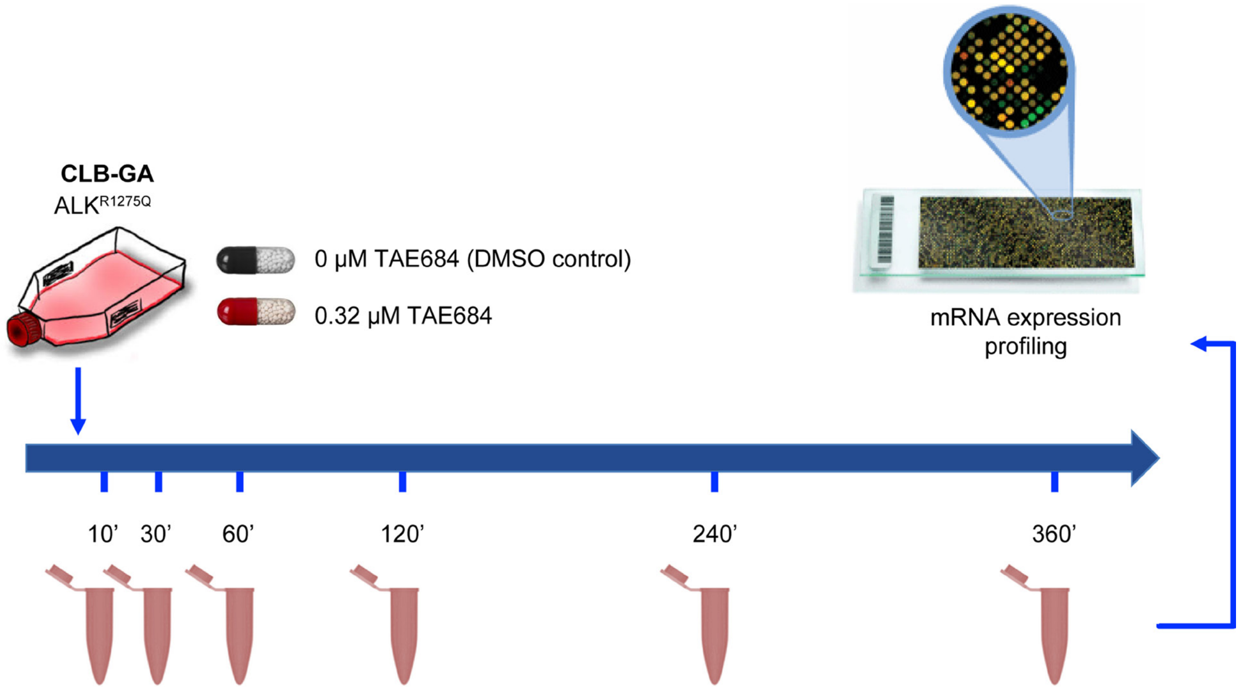

Supplementary Figure 1: Workflow to generate the time course dataset upon pharmacological ALK inhibition. CLB-GA, an ALK^{R1275Q} mutated NB cell line, was treated in duplicate with 0.32 µM TAE684 or DMSO. Cells were collected for RNA 10 and 30 minutes, 1, 2, 4 and 6 hours after the treatment. RNA was extracted, quality was checked and samples were used for mRNA expression profiling with the Sureprint G3 human GE 8x60K microarrays (Agilent Technologies).

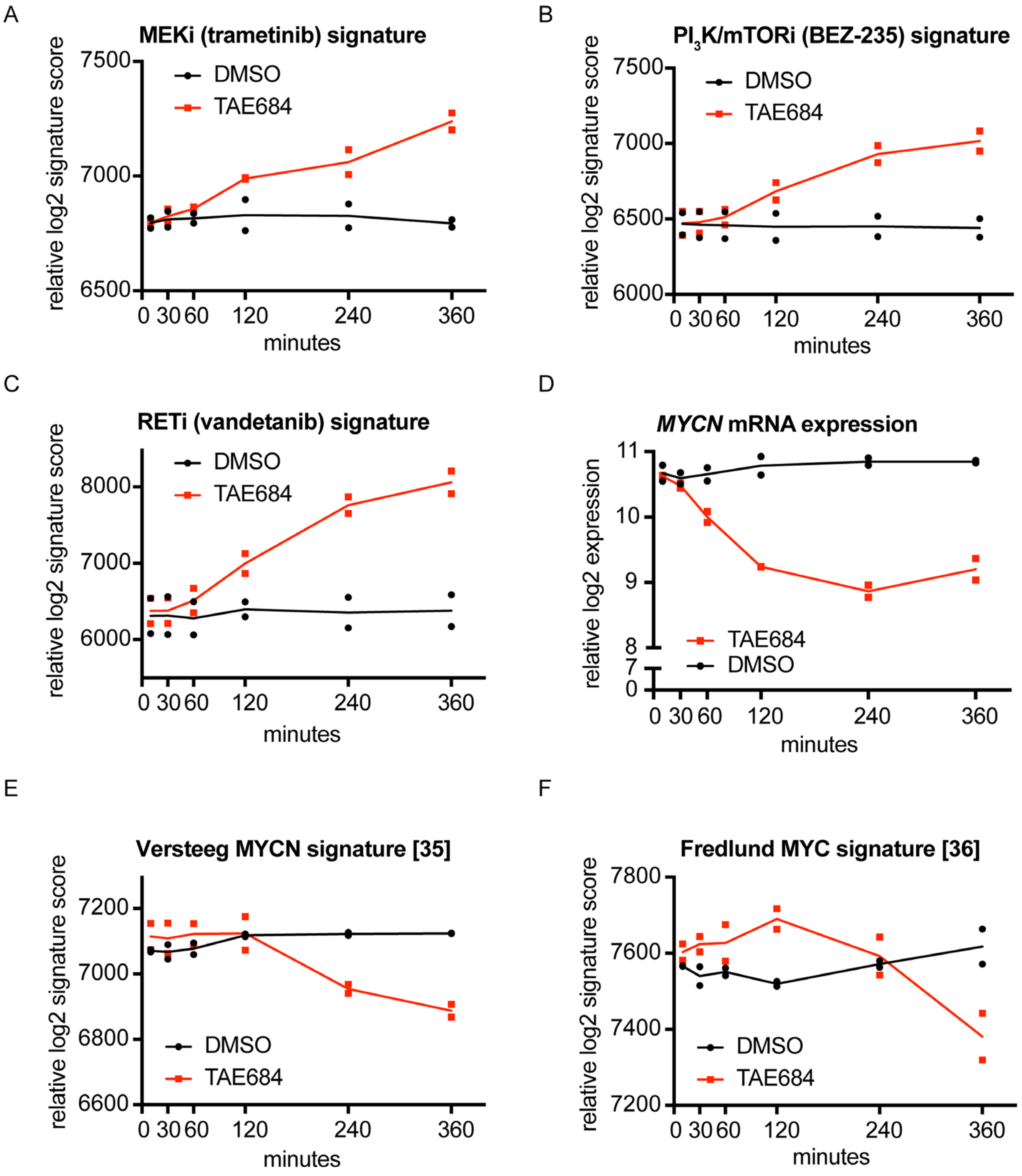

Supplementary Figure 2: Signature score analysis for MAPK, PI_3K, RET and MYC(N) signaling pathways, downregulated starting from 1 or 2 hours after TAE684 treatment (absolute values). (**A–F**) MEK inhibitor (trametinib) (A), PI_3K/mTOR inhibitor (BEZ-235) (B) and RET inhibitor (vandetanib) (C) signatures are upregulated from 1 or 2 hours after treatment of the CLB-GA cell line with 320 nM TAE684, while *MYCN* mRNA expression levels (D) are downregulated from 1 hour after treatment and the MYCN activity score from Valentijn *et al.* [35] (E) and the MYC signature score from Fredlund *et al.* [36] (F) from respectively 2 and 4 hours after pharmacological ALK blockade. Log2 transformed expression levels of TAE684 treated and DMSO control samples are plotted.

A

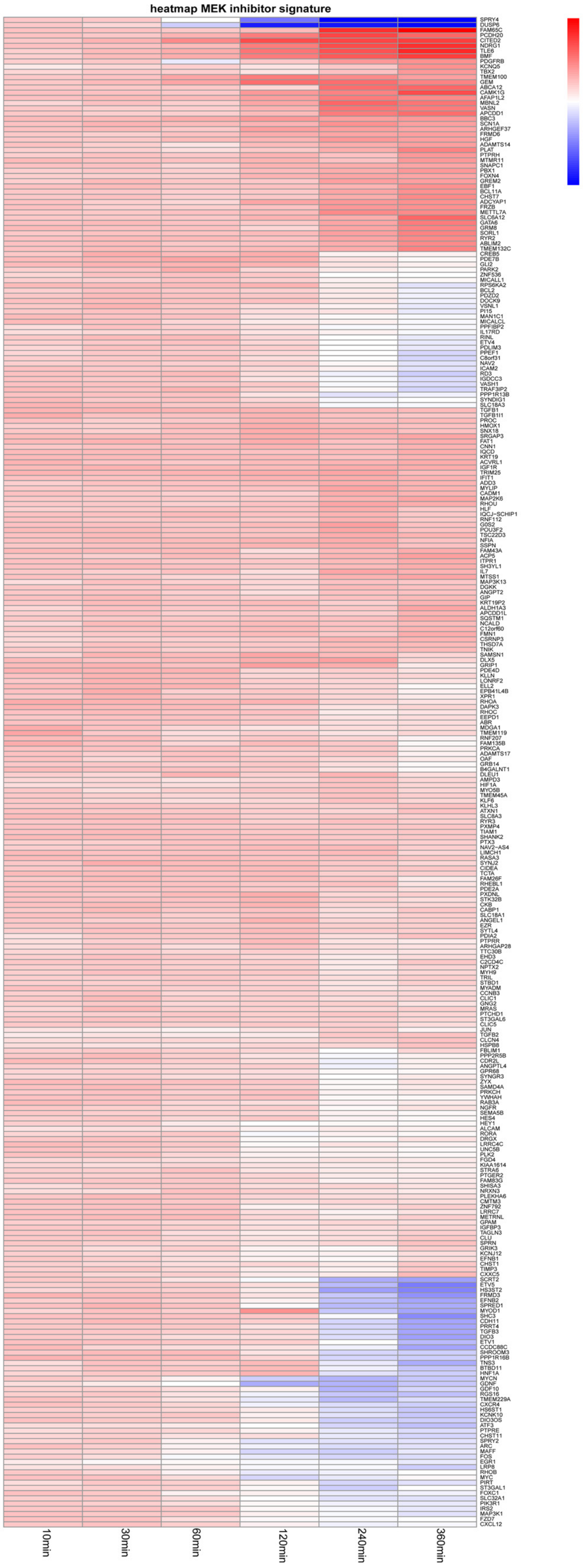
heatmap MEK inhibitor signature
2
1
0
−1
−2
−3
−4
SPRY4
DUSP6
FAM65C
PCDH20
CITED2
NDRG1
TLE6
BMF
PDGFRB
KCNG5
TBX2
TMEM100
GEM
ABCA12
CAMK1G
AFAP1L2
MBNL2
VASN
APCDD1
BBC3
SCN1A
ARHGEF37
FRMD6
HGF
ADAMTS14
PLAT
PTPRH
MTMR11
SNAPC1
PBX1
FOXN4
GREM2
EBF1
BCL11A
CHST7
ADCYAP1
FRZB
METTL7A
SLC6A12
GATA6
GRM8
SORL1
RYR2
ABLIM2
TMEM132C
CREB5
PDE7B
GLI2
DIRK2
ZNF536
MICALL1
RPS6KA2
BCL2
PDZD2
DOCK9
VSNL1
PI15
MAN1C1
MICALCL
PPFIBP2
IL17RD
RNL
ETV4
PDLIM3
PPEF1
C8orf31
NAV2
ICAM2
RD3
IGDCC3
VASH1
TRAF3IP2
PPP1R13B
SYNDIG1
SLC18A3
TGFB1
TGFB1I1
PROC
HMOX1
SNX18
SRGAP3
FAT1
CNN1
IQCD
KRT19
ACVRL1
IGF1R
TRIM25
IFIT1
ADD3
MYLIP
CADM1
MAP2K6
RHOU
HLF
IQCJ-SCHIP1
RNF112
G0S2
POU3F2
TSC22D3
NFIA
SSPN
FAM43A
ACP5
ITPR1
SH3YL1
IL7
MTSS1
MAP3K13
DGKK
ANGPT2
GIP
KRT19P2
ALDH1A3
APCDD1L
SQSTM1
NCALD
C12orf60
FMN1
CSRNP3
THSD7A
TNIK
SAMSN1
DLX5
GRIP1
PDE4D
KLLN
LONRF2
ELL2
EPB41L4B
XIRP1
RHOA
DAPK3
RHOC
EEPD1
ABR
MDGA1
TMEM119
RNF207
FAM135B
PRKCA
ADAMTS17
OAF
GRB14
B4GALNT1
DLEU1
AMPD3
HIF1A
MYO5B
TMEM45A
KLF6
KLHL3
ATXN1
SLC8A3
RYR3
PXMP4
TIAM1
PTX3
SHANK2
NAV2-AS4
LIMCH1
RASA3
SYNJ2
CIDEA
TCTA
FAM26F
RHEBL1
PDE2A
PXDNL
STK32B
CKB
CABP1
SLC18A1
ANGEL1
EZR
SYTL4
PDIA2
PTPRR
ARHGAP28
TTC30B
EHD3
C2CD4C
NPTX2
MYH9
TRIL
STBD1
MYADM
CCNB3
CLIC1
GNG2
MRAS
PTCHD1
ST3GAL6
CLIC5
JUN
TGFB2
CLCN4
HSPB8
FBLIM1
PPP2R5B
CDR2L
ANGPTL4
GPR68
SYNGR3
ZYX
SAMD4A
PRKCH
YWHAH
RAB3A
NGFR
SEMA5B
HES4
HEY1
ALCAM
RORA
DRGX
LRRC4C
UNC5B
PLK2
FGD4
KIAA1614
STRA6
PTGER2
FAM83G
SHISA3
NRXN3
PLEKHA6
CMTM3
ZNF792
LRRC7
METRNL
GPAM
IGFBP3
TAGLN3
CLU
SPRN
GRIK3
KCNJ12
EFNB1
CHST1
TIMP3
CXXC5
SCRT2
ETV5
HS3ST2
FRMD3
EFNB2
SPRED1
MYOD1
SHC3
CDH11
PRRT4
TGFB3
DIO3
ETV1
CCDC88C
SHROOM3
PPP1R16B
TNS3
BTBD11
HNF1A
MYCN
GDNF
GDF10
RGS16
TMEM229A
CXCR4
HS6ST1
KCNK10
DIO3OS
ATF3
PTPRE
CHST11
SPRY2
ARC
MAFF
FOS
EGR1
LRP8
RHOB
MYC
PIRT
ST3GAL1
FOXC1
SLC2A1
PIK3R1
IRS2
MAP3K1
FZD7
CXCL12
10min
30min
60min
120min
240min
360min

B

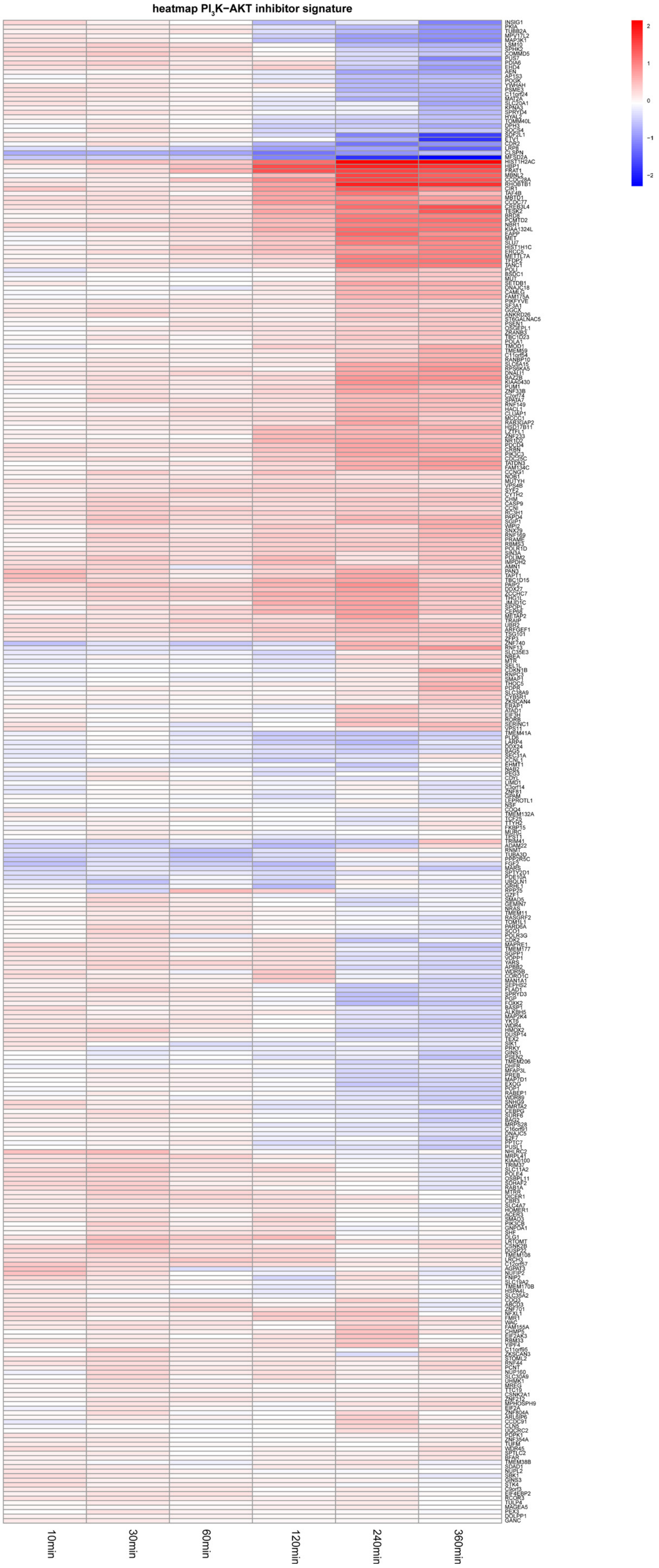

heatmap PI3K−AKT inhibitor signature
10min
30min
60min
120min
240min
360min
2
1
0
−1
−2

C

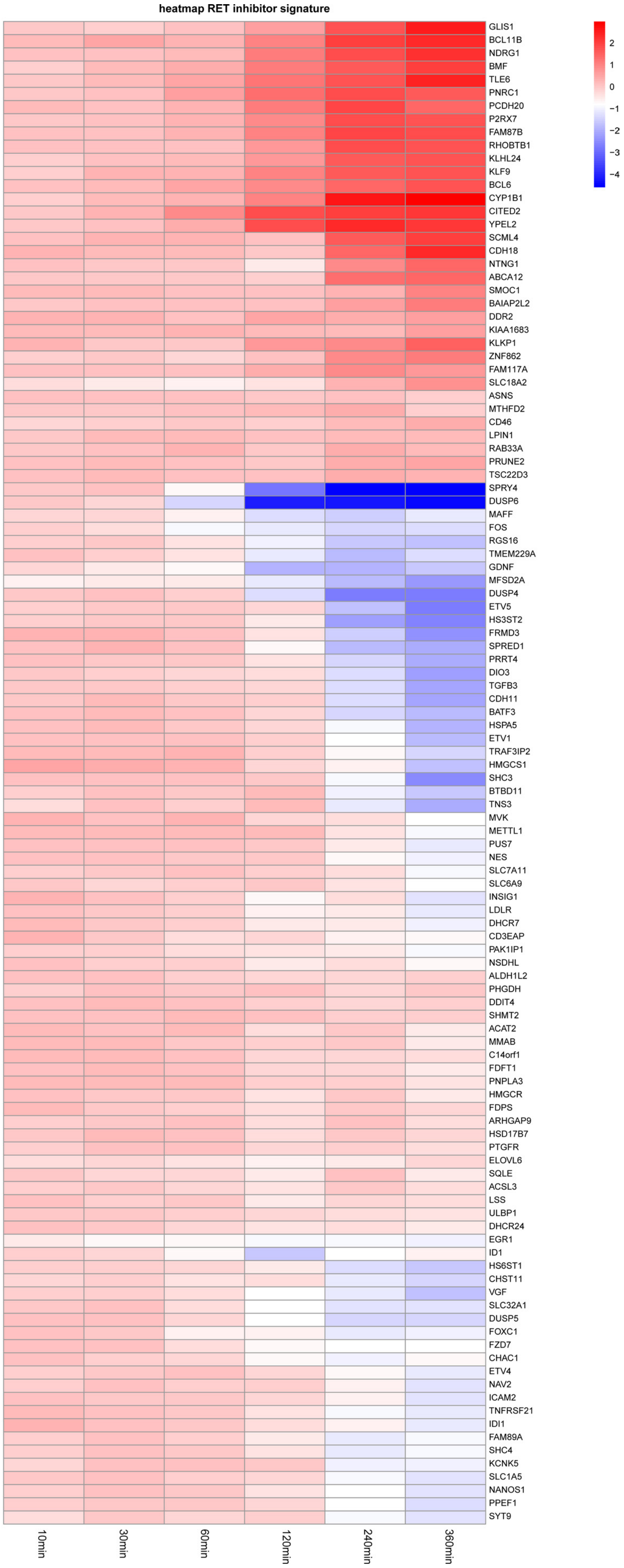
heatmap RET inhibitor signature
GLIS1
BCL11B
NDRG1
BMF
TLE6
PNRC1
PCDH20
P2RX7
FAM87B
RHOBTB1
KLHL24
KLF9
BCL6
CYP1B1
CITED2
YPEL2
SCML4
CDH18
NTNG1
ABCA12
SMOC1
BAIAP2L2
DDR2
KIAA1683
KLKP1
ZNF862
FAM117A
SLC18A2
ASNS
MTHFD2
CD46
LPIN1
RAB33A
PRUNE2
TSC22D3
SPRY4
DUSP6
MAFF
FOS
RGS16
TMEM229A
GDNF
MFSD2A
DUSP4
ETV5
HS3ST2
FRMD3
SPRED1
PRRT4
DIO3
TGFB3
CDH11
BATF3
HSPA5
ETV1
TRAF3IP2
HMGCS1
SHC3
BTBD11
TNS3
MVK
METTL1
PUS7
NES
SLC7A11
SLC6A9
INSIG1
LDLR
DHCR7
CD3EAP
PAK1IP1
NSDHL
ALDH1L2
PHGDH
DDIT4
SHMT2
ACAT2
MMAB
C14orf1
FDFT1
PNPLA3
HMGCR
FDPS
ARHGAP9
HSD17B7
PTGFR
ELOVL6
SQLE
ACSL3
LSS
ULBP1
DHCR24
EGR1
ID1
HS6ST1
CHST11
VGF
SLC32A1
DUSP5
FOXC1
FZD7
CHAC1
ETV4
NAV2
ICAM2
TNFRSF21
IDI1
FAM89A
SHC4
KCNK5
SLC1A5
NANOS1
PPEF1
SYT9
2
1
0
−1
−2
−3
−4
10min
30min
60min
120min
240min
360min

D

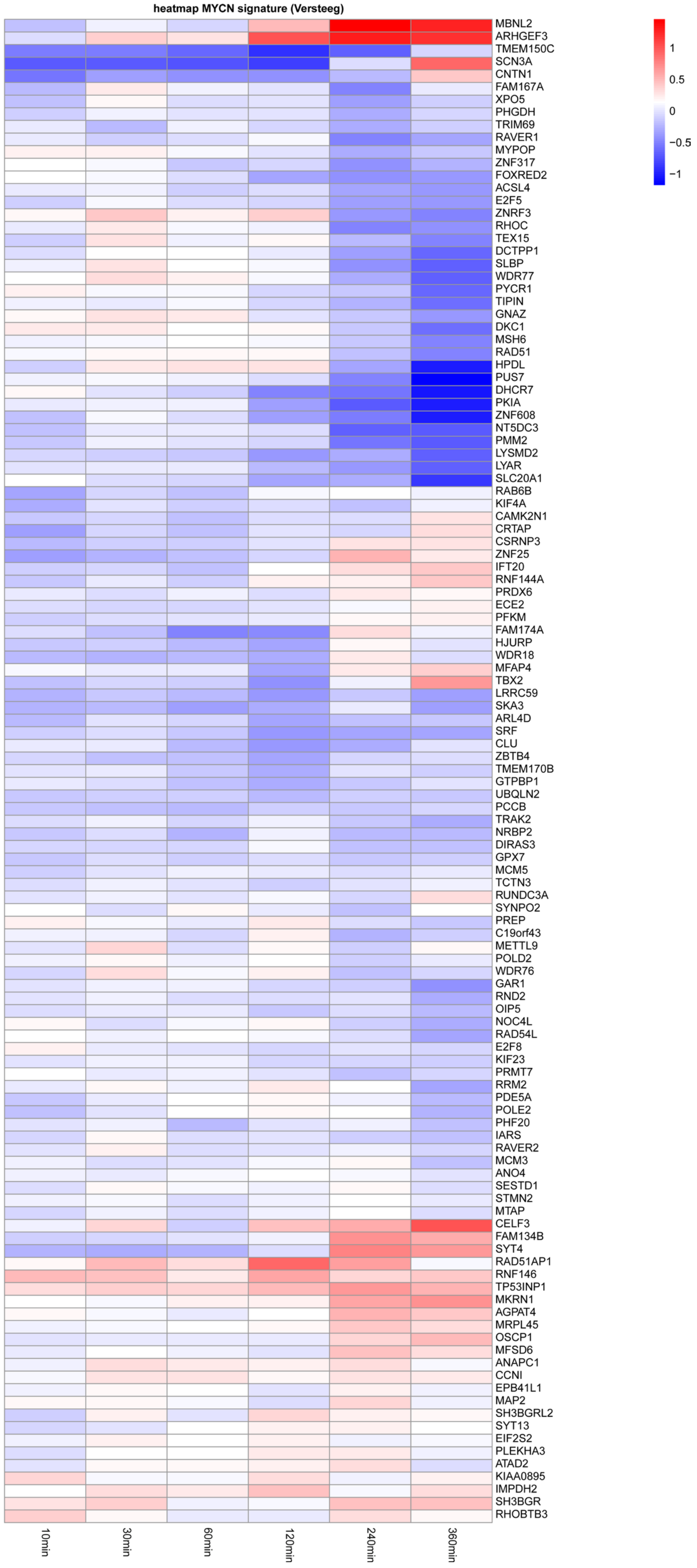
heatmap MYCN signature (Versteeg)
MBNL2
ARHGEF3
TMEM150C
SCN3A
CNTN1
FAM167A
XPO5
PHGDH
TRIM69
RAVER1
MYPOP
ZNF317
FOXRED2
ACSL4
E2F5
ZNRF3
RHOC
TEX15
DCTPP1
SLBP
WDR77
PYCR1
TIPIN
GNAZ
DKC1
MSH6
RAD51
HPDL
PUS7
DHCR7
PKIA
ZNF608
NT5DC3
PMM2
LYSMD2
LYAR
SLC20A1
RAB6B
KIF4A
CAMK2N1
CRTAP
CSRNP3
ZNF25
IFT20
RNF144A
PRDX6
ECE2
PFKM
FAM174A
HJURP
WDR18
MFAP4
TBX2
LRRC59
SKA3
ARL4D
SRF
CLU
ZBTB4
TMEM170B
GTPBP1
UBQLN2
PCCB
TRAK2
NRBP2
DIRAS3
GPX7
MCM5
TCTN3
RUNDC3A
SYNPO2
PREP
C19orf43
METTL9
POLD2
WDR76
GAR1
RND2
OIP5
NOC4L
RAD54L
E2F8
KIF23
PRMT7
RRM2
PDE5A
POLE2
PHF20
IARS
RAVER2
MCM3
ANO4
SESTD1
STMN2
MTAP
CELF3
FAM134B
SYT4
RAD51AP1
RNF146
TP53INP1
MKRN1
AGPAT4
MRPL45
OSCP1
MFSD6
ANAPC1
CCNI
EPB41L1
MAP2
SH3BGRL2
SYT13
EIF2S2
PLEKHA3
ATAD2
KIAA0895
IMPDH2
SH3BGR
RHOBTB3
10min
30min
60min
120min
240min
360min
1
0.5
0
−0.5
−1

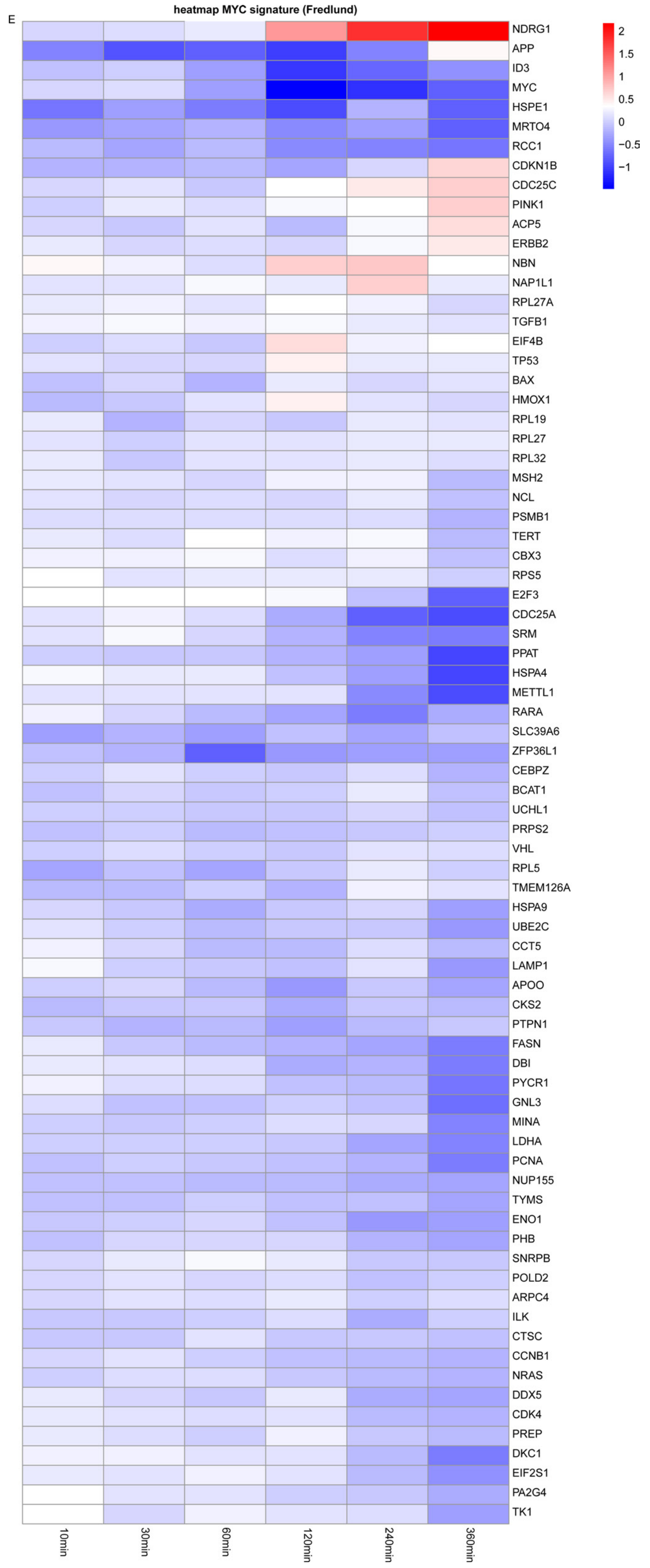

Supplementary Figure 3: Heatmaps representing the genes from the signature score analysis for MAPK, PI_3K, RET and MYC(N) signaling pathways. (**A–E**) Heatmaps showing the dynamic expression over time for the genes from the MEK inhibitor (trametinib) (A), the PI_3K-AKT inhibitor (B), the RET inhibitor (vandetanib) (C), the Versteeg MYCN [35] signature (D) and the Fredlund MYC [36] signature (E). The mean of the ratio of the expression in the TAE684 vs DMSO treated sample is plotted for each gene over time.

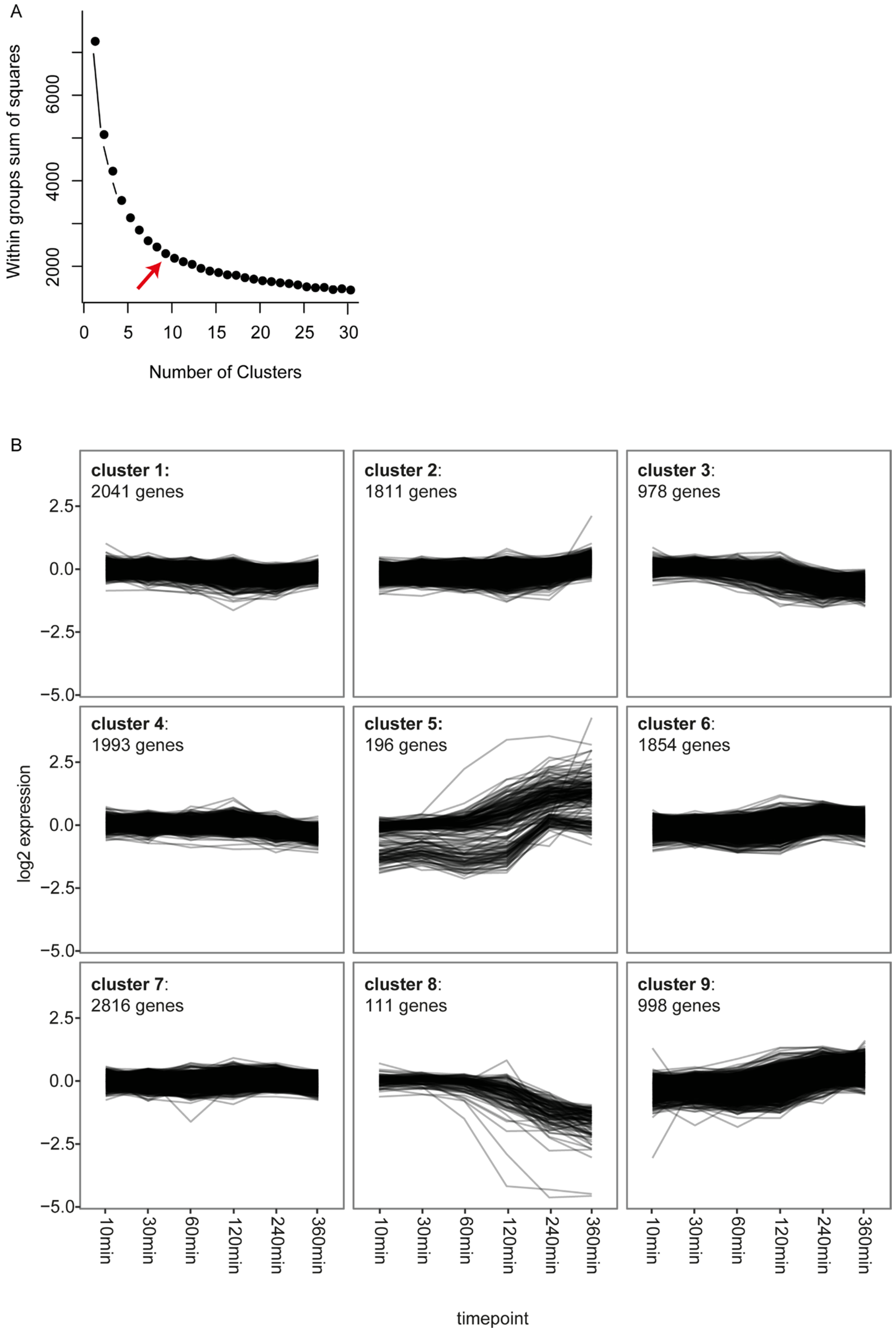

Supplementary Figure 4: K-means clustering of the expression data. (**A**) Plot illustrating the choice to perform k-means clustering with k = 9 (red arrow) as determined by the elbow method. The y-axis shows the variability within the groups and the x-axis the number of clusters. (**B**) The cluster plots represent the dynamic pattern of the expression of the genes belonging to 1 of the 9 clusters. The mean of the ratio of the expression in the TAE684 vs DMSO treated sample is plotted for each gene.

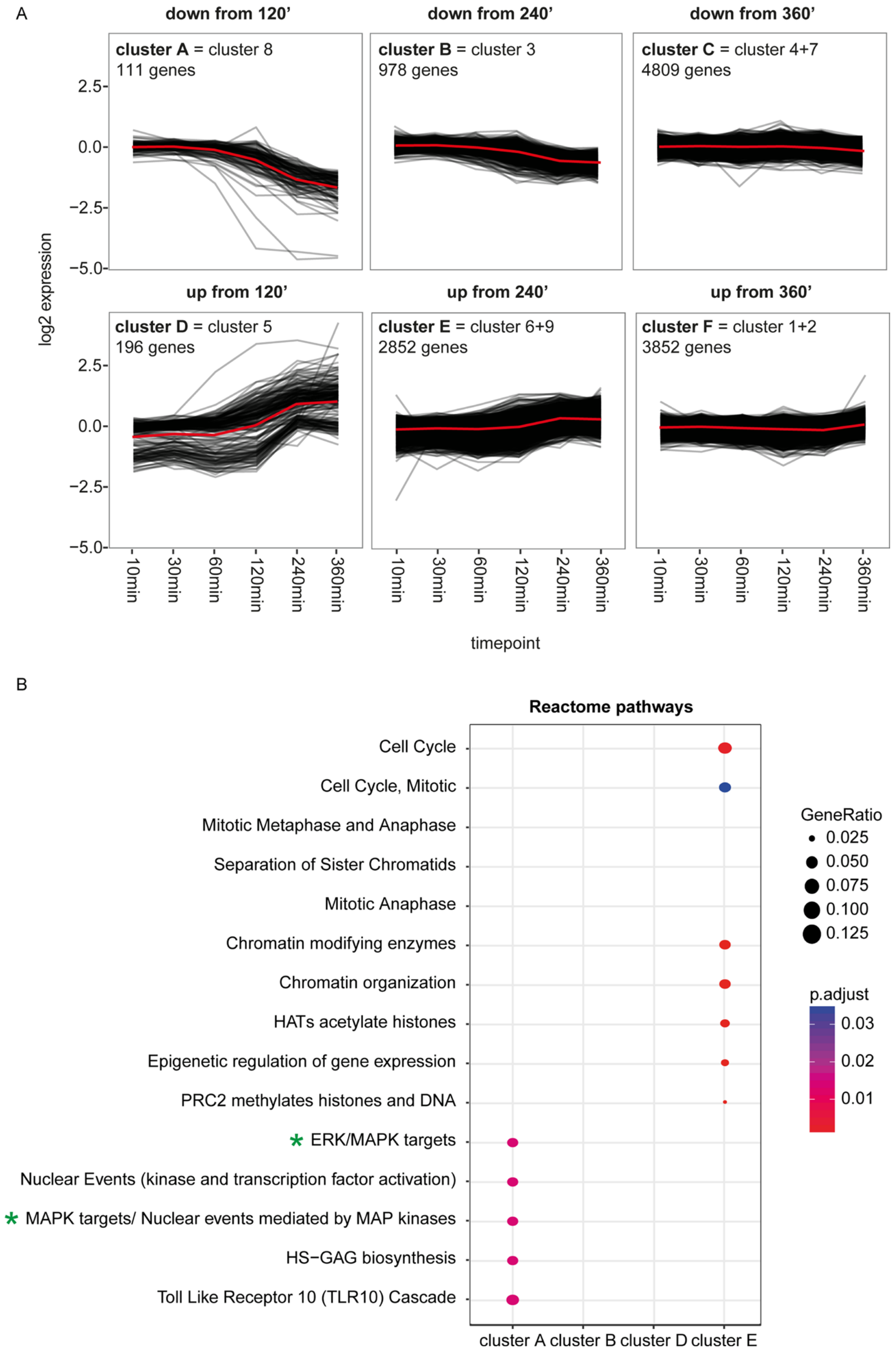

A
down from 120’
down from 240’
down from 360’
cluster A = cluster 8
111 genes
cluster B = cluster 3
978 genes
cluster C = cluster 4+7
4809 genes
up from 120’
up from 240’
up from 360’
cluster D = cluster 5
196 genes
cluster E = cluster 6+9
2852 genes
cluster F = cluster 1+2
3852 genes
log2 expression
2.5
0.0
−2.5
−5.0
10min
30min
60min
120min
240min
360min
timepoint
B
Reactome pathways
Cell Cycle
Cell Cycle, Mitotic
Mitotic Metaphase and Anaphase
Separation of Sister Chromatids
Mitotic Anaphase
Chromatin modifying enzymes
Chromatin organization
HATs acetylate histones
Epigenetic regulation of gene expression
PRC2 methylates histones and DNA
* ERK/MAPK targets
Nuclear Events (kinase and transcription factor activation)
* MAPK targets/ Nuclear events mediated by MAP kinases
HS−GAG biosynthesis
Toll Like Receptor 10 (TLR10) Cascade
cluster A cluster B cluster D cluster E
GeneRatio
0.025
0.050
0.075
0.100
0.125
p.adjust
0.03
0.02
0.01

C

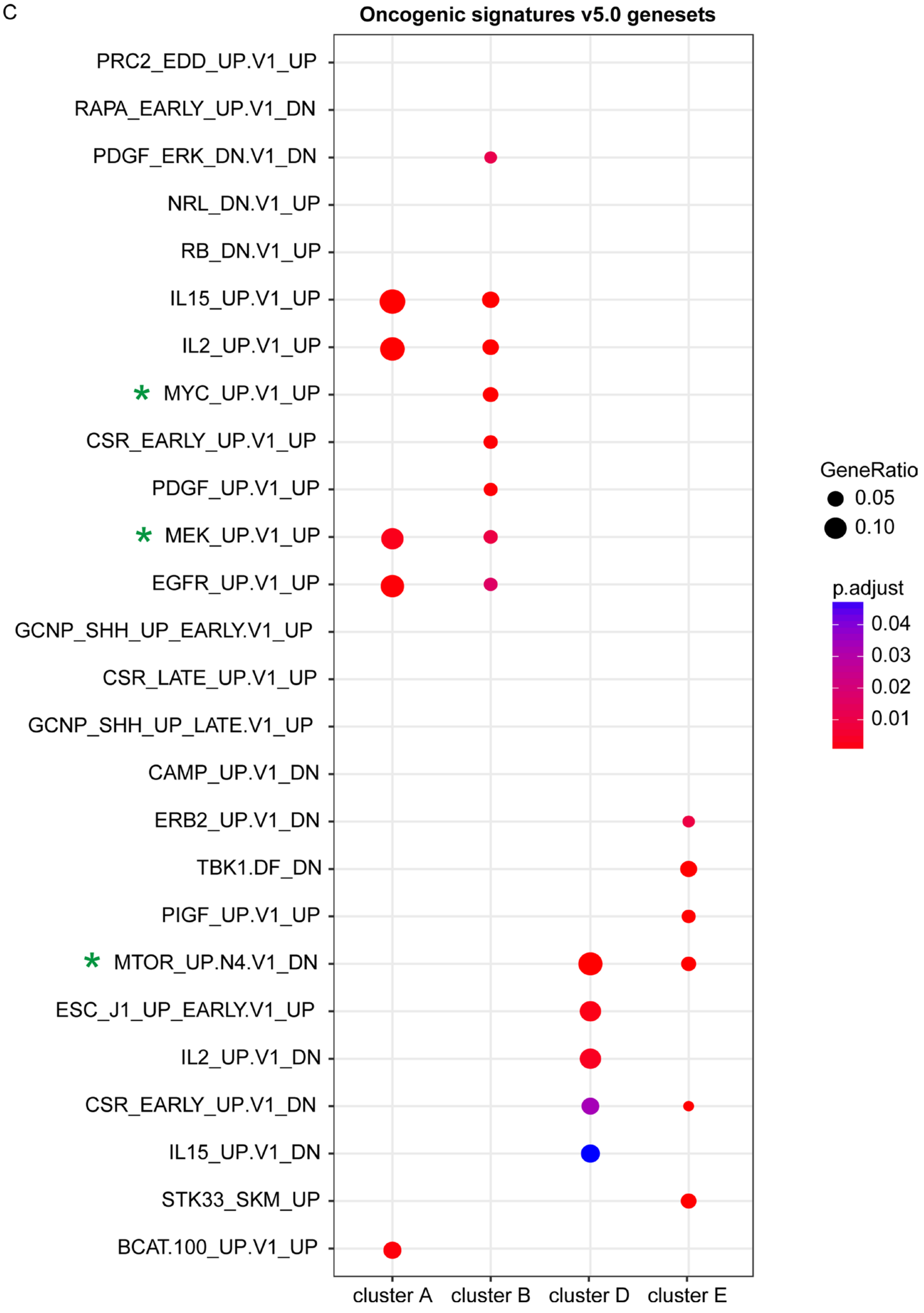

Supplementary Figure 5: Functional characterization of the genes in the clusters with the Reactome Pathways and MSigDB 'c6 Oncogenic Signatures v5.0'. (**A**) The cluster plots represent the dynamic pattern of the expression of the genes belonging to 1 of the 6 clusters. The mean of the ratio of the expression in the TAE684 vs DMSO treated sample is plotted for each gene. The red lines show the average dynamic pattern of the expression of the genes belonging to these clusters, calculated by the average of the mean of the ratio of the expression in the TAE684 vs DMSO treated sample. (**B**) The plot shown pathways of the Reactome Database that are enriched in at least one of the four clusters, which are showing a clear dynamic pattern (clusters A, B, D, E). The size of each node corresponds to the number of genes overlapping between the cluster and the gene set and the colour represents the adjusted p-value of the enrichment test. (**C**) The plot shown the MSigDB 'c6 Oncogenic Signatures v5.0' genesets that are enriched in at least one of the four clusters, which are showing a clear dynamic pattern (clusters A, B, D, E). The size of each node corresponds to the number of genes overlapping between the cluster and the gene set and the colour represents the adjusted p-value of the enrichment test. Green stars indicate the genesets related to the MYC(N), KRAS-MAPK, PI_3K/mTOR pathways.

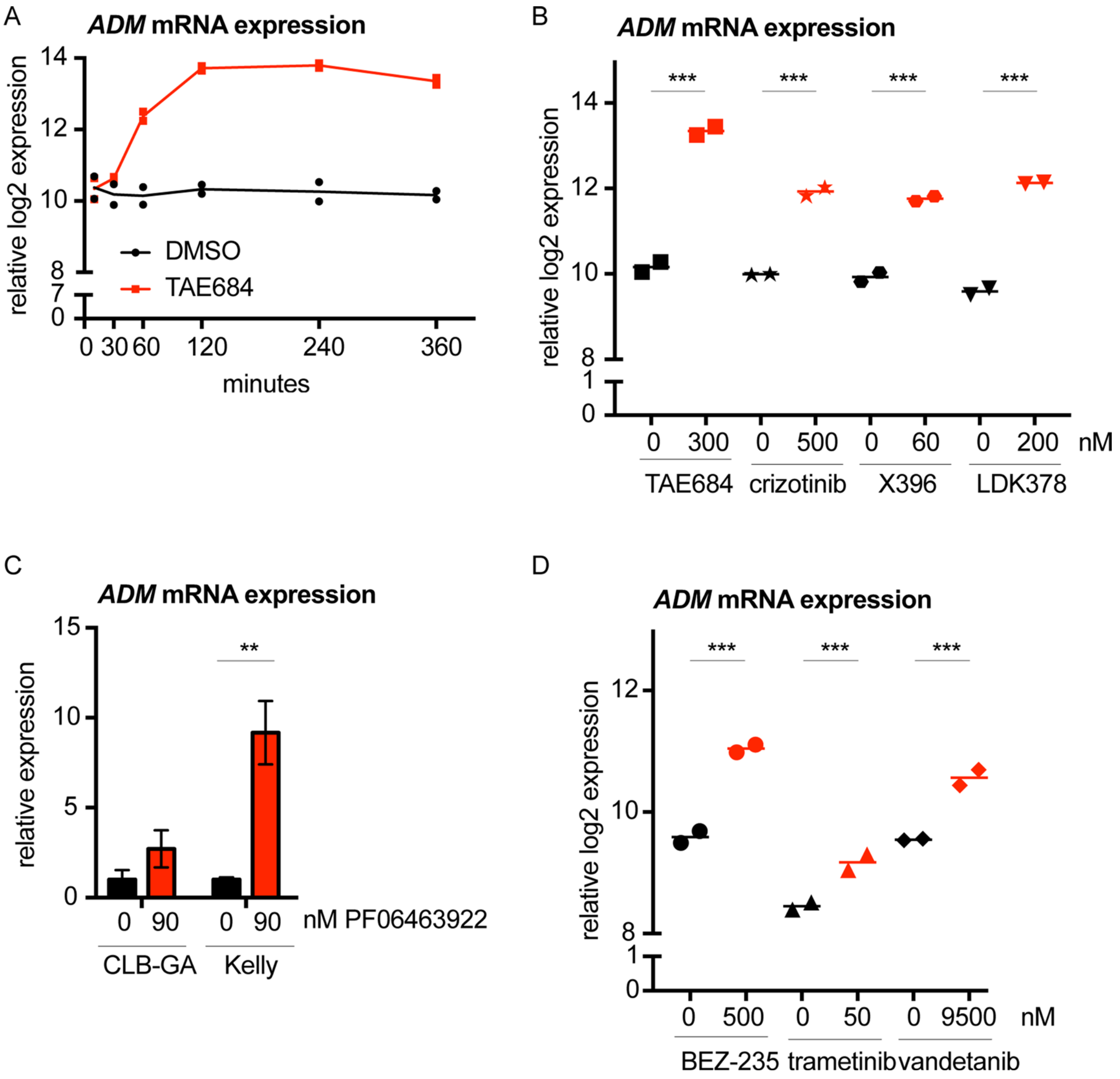

Supplementary Figure 6: ADM expression upon pharmacological blockade of ALK and ALK downstream signaling in NB cells. (**A**) *ADM* mRNA is significantly upregulated starting from 1 hour after TAE684 treatment of the CLB-GA cell line. Log2 transformed expression levels of TAE684 treated and DMSO control samples are plotted. (**B**) *ADM* mRNA expression levels are increased in CLB-GA treated for 6 h with 0.32 μM TAE684, 0.5 μM crizotinib, 0.06 μM X396, 0.2 μM LDK378 compared to the DMSO control. (**C**) *ADM* mRNA expression levels are upregulated in NB cell lines CLB-GA (ALK^{R1275Q}) and Kelly (ALK^{F1174L}) treated for 6 hours with 0.09 μM PF06463922 (new-generation ALK inhibitor) compared to the DMSO control. (**D**) *ADM* mRNA expression levels are increased in CLB-GA treated with 0.5 μM BEZ-235, 0.05 μM trametinib, 9.5 μM vandetanib compared to DMSO for 6 h. Statistical analyses: unpaired one-way ANOVA with Bonferroni correction (B. & C. & D.). $^{*}P < 0.05$, $^{**}P < 0.01$, $^{***}P < 0.001$.

Supplementary Table 1: Significantly, differentially expressed genes (excel). Supplementary data table listing the significantly differentially expressed genes upon ALK inhibition at every time point (FDR < 0.001). The first sheet is a summary of the significantly differentially expressed genes, the second sheet contains the genes upregulated upon ALK inhibition and the third sheet shows the downregulated genes. See Supplementary_Table_1

Supplementary Table 2: Primer sequences

Gene	Primer	Sequence
ADM	Forward	GAATCCGAGTGTTTGCCAGG
	Reverse	ACACGCATTGCACTTTTCCT
TBP	Forward	CACGAACCACGGCACTGATT
	Reverse	TTTTCTTGCTGCCAGTCTGGAC
YWHAZ	Forward	ACTTTTGGTACATTGTGGCTTCAA
	Reverse	CCGCCAGGACAAACCAGTAT
SDHA	Forward	TGGGAACAAGAGGGCATCTG
	Reverse	CCACCACTGCATCAAATTCATG
B2M	Forward	TGCTGTCTCCATGTTTGATGTATCT
	Reverse	TCTCTGCTCCCCACCTCTAAGT
HPRT1	Forward	TGACACTGGCAAAACAATGCA
	Reverse	GGTCCTTTTCACCAGCAAGCT

Primer sequences used for qPCR expression analysis of the *ADM* gene and 5 reference genes.
